# ﻿Rodents of Chile: a brief appraisal of their conservation status and ecological significance

**DOI:** 10.3897/zookeys.1254.148057

**Published:** 2025-10-01

**Authors:** Wendy C. Hernández-Mazariegos, R. Eduardo Palma, Luis E. Escobar

**Affiliations:** 1 Programa de Doctorado en Medicina de la Conservación, Facultad de Ciencias de Vida, Universidad Andres Bello, Avenida República 440, Santiago 8370251, Chile Universidad Andres Bello Santiago Chile; 2 Facultad de Ciencias Biológicas, Pontificia Universidad Católica de Chile, Av. Libertador Bernardo O’Higgins 340, Santiago 8331150, Chile Pontificia Universidad Católica de Chile Santiago Chile; 3 Universidad de O’Higgins, Avenida Libertador Bernardo O’Higgins 611, Rancagua, 2841959, Chile Universidad de O’Higgins Rancagua Chile; 4 Department of Fish and Wildlife Conservation, Virginia Tech, Blacksburg, VA 24061, USA Virginia Tech Blacksburg United States of America; 5 Center for Emerging, Zoonotic and Arthropod-borne Pathogens, Virginia Tech, Blacksburg, VA 24060, USA Universidad Andres Bello Santiago Chile; 6 Global Change Center, Virginia Tech, Blacksburg, VA 24061, USA Pontificia Universidad Católica de Chile Santiago Chile; 7 The Kellogg Center for Philosophy, Politics, and Economics, Virginia Tech, Blacksburg, VA 24061, USA Universidad de O’Higgins Rancagua Chile; 8 One Health Institute, Faculty of Life Sciences, Universidad Andres Bello, Santiago, Chile Virginia Tech Blacksburg United States of America

**Keywords:** Biogeography, conservation, ecosystem services, public health, rodents, zoonoses

## Abstract

Rodentia is the most widely distributed, diverse, and numerous order of the class Mammalia. Nevertheless, rodents are poorly studied in terms of their conservation compared to other mammalian orders. Chile has one of the highest rates of extinction risk in the world for mammals (20%), where rodents have the highest risk (32%). The data of threatened rodent species is not comprehensive, as many species are still classified as data deficient. This lack of information could mean that the actual number of threatened species is higher than currently recognized. Using different databases, the biogeography, conservation status, ecological roles of rodent species in Chile are updated and described, and their potential zoonotic implication discussed. Results revealed that rodent species richness is highest in the northern and central-southern regions of Chile, where fewer protected areas exist, suggesting an inefficient role of public protected areas for the conservation of rodents and potentially other taxa. The conservation classification by the Chilean government did not match the conservation status from international classifications, revealing poor information for several species at national level. Functional traits of the species studied suggest that rodents are good predictors of ecosystem health due to their rapid life cycles and wide distribution, although distribution was predictive for only some species. Our results indicated that better information on the distribution and rodent species richness provide opportunities to understand complex rodent-borne diseases such as hantavirus. This study validates the use of rodents as indicators to assess ecosystem health and design effective biodiversity conservation plans.

## ﻿Introduction

Rodents (order Rodentia) comprise ~2500 extant species, representing nearly one-half (40%) of modern mammal species (Lacher et al. 2016; IUCN 2024a; MDD 2024). Despite this rich diversity, rodents have received less research attention compared with other mammalian orders (e.g., primates, pinnipeds, large carnivores), particularly from fields such as ecology and biological conservation (Gaulke et al. 2019; Kennerley et al. 2021). At least 324 (15%) rodents are threatened with extinction (Kennerley et al. 2021; Llobet et al. 2023), and more than 100 species are considered “evolutionarily distinct” and “globally endangered”, meaning that if one of these species disappear there would be no other species that replace it in an ecosystem (EDGE 2024). There are 452 rodent species classified as data deficient, which means that there are no data available to estimate their conservation status and they could actually be threatened (Entwistle and Stephenson 2000; Kennerley et al. 2021). In the last 500 years, of the nearly 100 species that have become extinct, it is reported that more than 50% are rodents (MacPhee and Flemming 1999; Turvey 2009; Teta et al. 2014). For example, at continental and insular level, in South and Central America, approximately 32 species of rodents disappeared in the last five centuries (MacPhee and Flemming 1999; Turvey 2009; Teta et al. 2014; Hadler et al. 2024). Together, these patterns suggest that rodents are under considerable extinction pressure and are a neglected group among mammals, contradicting the generalized perception that rodents lack conservation concern (MacPhee and Flemming 1999; Amori et al. 2016; Kennerley et al. 2021).

Mammal conservation is a priority in Chile, a country recognized as a global biodiversity hotspot, with 14% of mammals considered endemic but with one of the highest extinction rates of native mammals (20% of species) (Gaulke et al. 2019). Therefore, Chile is an excellent model for examining trends in species-specific biological and ecological information regarding conservation trajectories (Gaulke et al. 2019). Due to the fact that, over 50% of the habitat types have insufficient or no protection within public protected areas (Pauchard and Villarroel 2002), highlighting a bias in biodiversity protection.

In total, Chile hosts 166 species of native mammals (Palma and Rosende in press), of which > 40% are rodents (D'Elía et al. 2020; Iriarte 2021). Chilean rodents have diverse conservation challenges, including limited research (< 100 studies vs > 200 for large mammals; Gaulke et al. 2019), and potential poor protection, as their richness is clustered in the north (17–22 °S) and south-central (37–45 °S) parts of the country (Cofré and Marquet 1999; Cofré et al. 2007; Samaniego and Marquet 2009). Rodent distribution in Chile is likely inconsistent with the distribution of terrestrial protected areas along the country, which are mainly in the south (Marquet et al. 2019). Additionally, although rodents already face the highest risk of extinction (32%) of all mammalian orders in Chile (Gaulke et al. 2019; IUCN 2024a), this number may be an underestimate due to the large number of species with data deficient status (IUCN 2024a; MMA 2024). Worldwide, it has been estimated that more than 50% of data-poor rodents could be at risk of extinction (Jetz and Freckleton 2015), highlighting the importance of paying special attention to species with this classification.

In Chile, most research on threatened and endangered mammals, including rodents (e.g., *Ctenomys magellanicus*, *Octodon bridgesi*, *Octodon lunatus*) has primarily focused on aspects regarding their life history, while studies addressing threats and human dimensions remain scare (Gaulke et al. 2019). Similarly, ecosystem services and conservation risks of rodents have been poorly studied (Lacher et al. 2016). Therefore, the objective of this study was to summarize the state of knowledge of the biogeography, conservation status, and ecosystem services of rodents in Chile and propose future research lines.

## ﻿Materials and methods

### ﻿Data collection and conservation status

Data were collected on the seven rodent families present in Chile, including six families from the Caviomorpha group—Abrocomidae, Caviidae, Chinchillidae, Ctenomyidae, Echimyidae, and Octodontidae—and one family from the Sigmodontinae group, Cricetidae (D'Elía et al. 2020; Iriarte 2021). Rodent species included in this study followed the updated list of living mammals of Chile by D'Elía et al. (2020), as baseline. Nevertheless, recently reported taxonomic and nomenclature changes were considered. For instance, *Lagidium peruanum* was not included (Iriarte 2021; MDD 2024) and *Euneomys mordax* was actualized to *E. fossor* (Teta et al. 2021). The taxonomic and nomenclature changes were reviewed according to the “Mammal Diversity Database” (MDD 2024) (Suppl. material 1: table S1). Also, three additional species were included: *Oligoryzomys yatesi* (Palma and Rodríguez-Serrano 2018), *Punomys lemminus* (Quiroga-Carmona et al. 2023a) and *Oligoryzomys flavescens* (Quiroga-Carmona et al. 2023b).

Threat status classification by species was determined by the “Red List Classification” of the International Union for Conservation of Nature (IUCN 2024a), and “Regulations to Classify Species According to their Conservation Status” (RCE) of the government of Chile (MMA 2024) (Suppl. material 1: table S2). Species that did not have a threat classification by IUCN were classified as “Not Evaluated” following the “Guidelines for Using the IUCN Red List Categories and Criteria” (IUCN Standards and Petitions Committee 2024b). Also, the main threats by rodent species were categorized according to the IUCN (2024a) threat classification.

### ﻿Species richness, conservation, and Protected Terrestrial areas

Distribution ranges of the species studied were downloaded from the IUCN (2024a) and Map of Life (Jetz et al. 2012) as shapefiles to explore the richness and distribution of the rodents of Chile (Suppl. material 1: table S1). Maps of both richness and threatened species were constructed using R software version 2024.09.0 (R Core Team 2024) with *sf* (Pebesma and Bivand 2023) and *raster* (Hijmans et al. 2023) packages, and cartography was developed using QGIS v. 3.38.3 (QGIS Development Team 2024). The spatial layer of Protected Terrestrial Areas (TPAs) was downloaded from National Congress Library of Chile (BCN 2024) and edited in QGIS.

### ﻿Species traits

Trait data were obtained from different databases, including AnimalTraits (Herberstein et al. 2022), AnAge (De Magalhães and Costa 2009), PanTHERIA (Jones et al. 2009), COMBINE (Soria et al. 2021), Elton Traits (Wilman et al. 2014), and IUCN (2024a). Traits assessed included body mass, body length, gestation length, litter size, litter size per year, sexual maturity, weaning length, and distribution range size. Traits were combined (e.g., summing the values ​​of a trait found in different sources for a species) and the average value for each trait was used for analysis. A principal components analysis (PCA) was performed with the trait data to evaluate the ecological linkages among species (Jolliffe 2002). The analysis was performed in R (R Core Team 2024), using the packages *factoextra* (Kassambara and Mundt 2020) and *car* (Fox et al. 2024). Only the rodent species that had trait data were included (Suppl. material 1: table S4).

## ﻿Results

**Figure 1.**
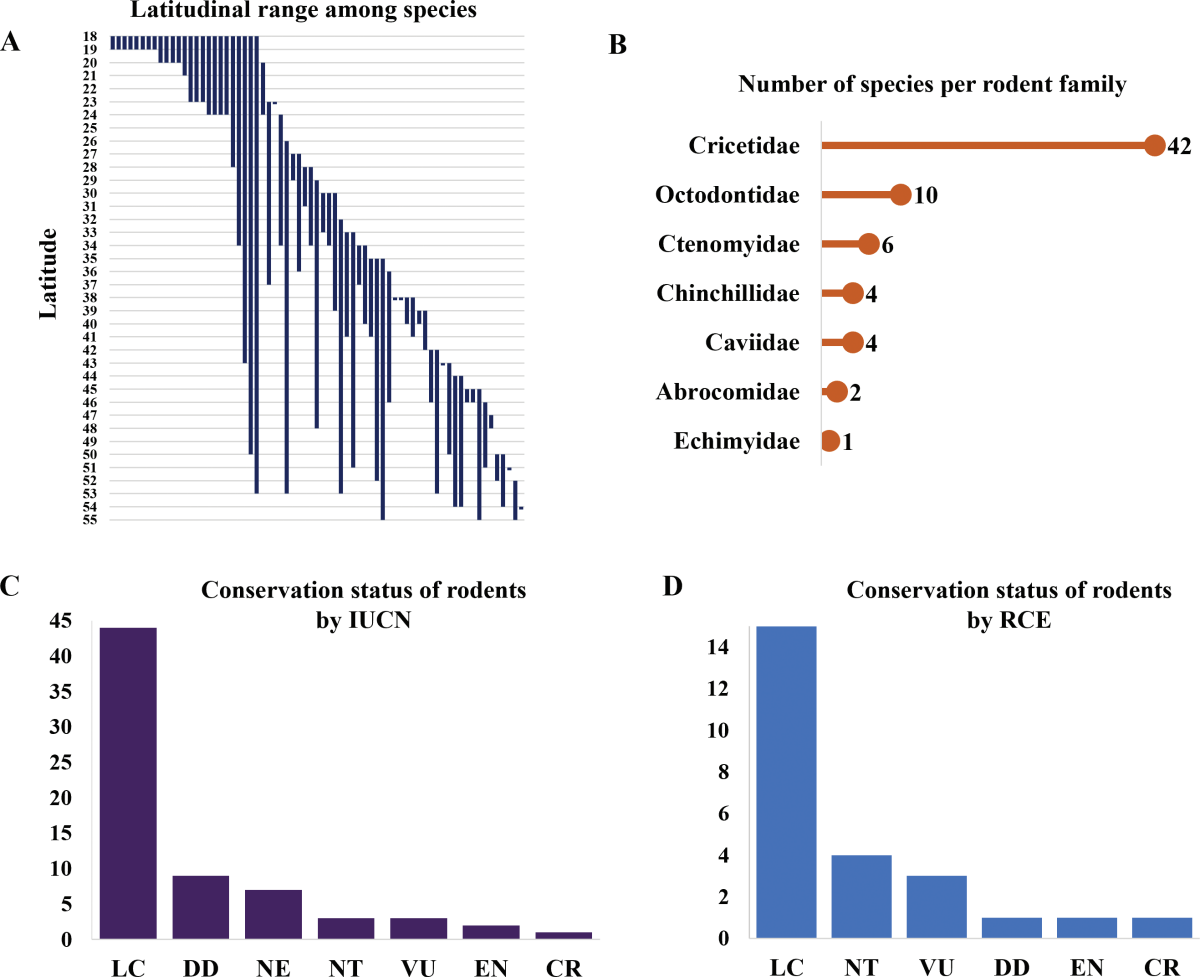
Distribution and conservation of rodents of Chile according to taxa. **A.** Latitudinal ranges of 69 rodent species of Chile; **B.** Number of rodent species per family; **C.** Conservation status of rodents by IUCN (2024a). DD: Data Deficient, NE: Not Evaluated, LC: Least Concern, NT: Near Threatened, VU: Vulnerable, EN: Endangered, CR: Critically Endangered (IUCN 2024a); **D.** Conservation status of rodents by "Regulations to Classify Species According to their Conservation Status" (RCE) of the government of Chile (MMA 2024) DD: Data Deficient, Least Concern, NT: Near Threatened, VU: Vulnerable, EN: Endangered, CR: Critically Endangered.

Family Cricetidae had the largest number of rodent species (*n* = 42), followed by Octodontidae (*n* = 10), Ctenomyidae (*n* = 6), Chinchillidae (*n* = 4), Caviidae (*n* = 4), Abrocomidae (*n* = 2), and Echimyidae (*n* = 1) (Fig. 1; Suppl. material 1: table S1). According to the IUCN classification, 44 rodent species were classified as “least concern”, nine in a risk category (i.e., “near threatened”, “vulnerable”, “endangered”, “critically endangered”), nine as “data deficient” and seven as “not evaluated” (Fig. 1; Suppl. material 1: table S2). Following “Regulations to Classify Species According to their Conservation Status” (MMA 2024) there was information to classify only 25 species categorized for Chile, of which 15 were classified as “least concern”, four as “near threatened”, three as “vulnerable”, one as “endangered”, one as “critically endangered”, and one as “data deficient” (Fig. 1, Suppl. material 1: table S2).

**Figure 2.**
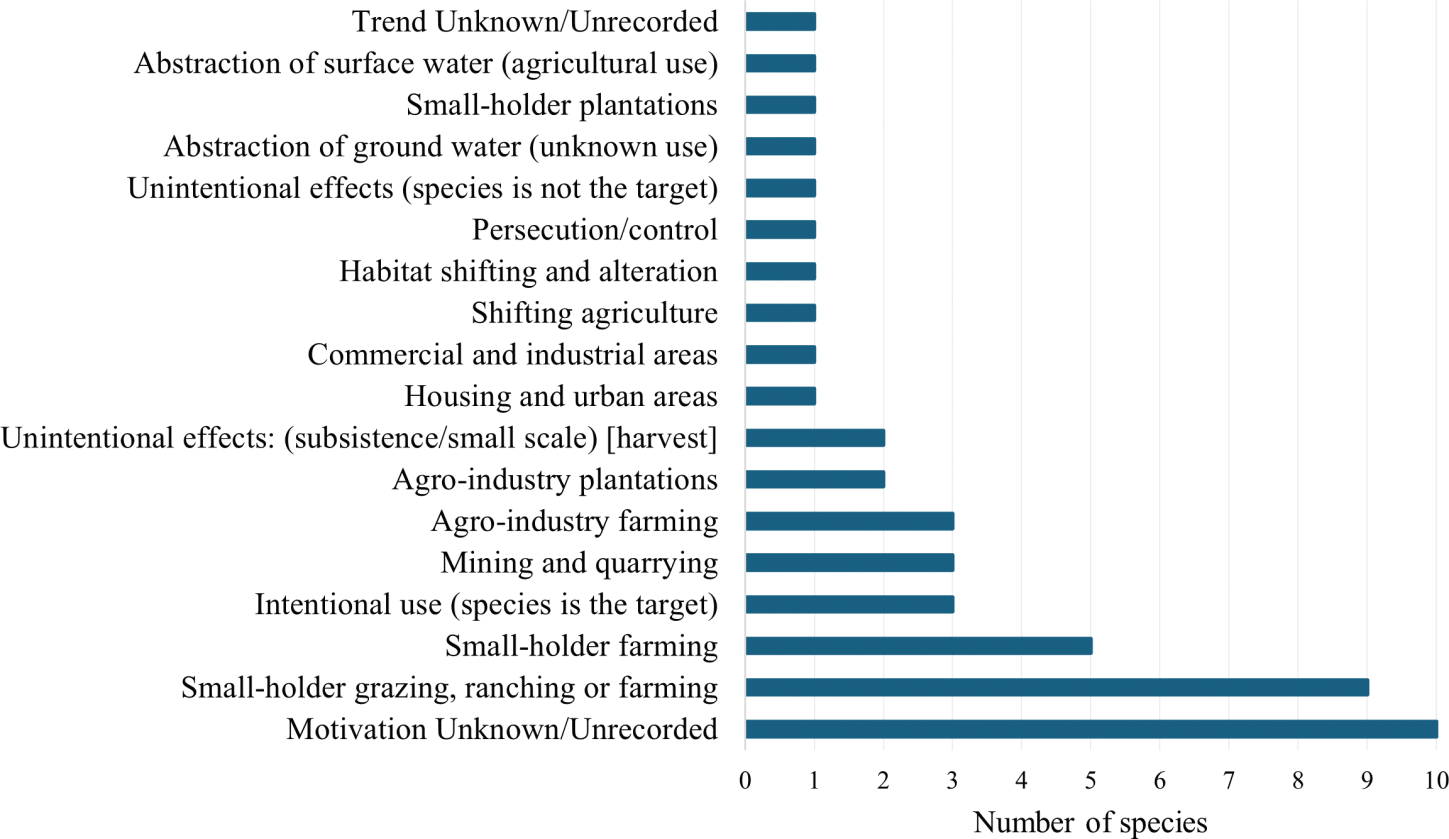
Main threats of rodents of Chile.

According to IUCN (2024a), 18 types of threats are recorded for rodents of Chile, which are mainly caused by biological resources use, agriculture, energy production and mining, and natural modification system (Fig. 2). At least 22 species presented one type of threat (Suppl. material 1: table S3).

**Figure 3.**
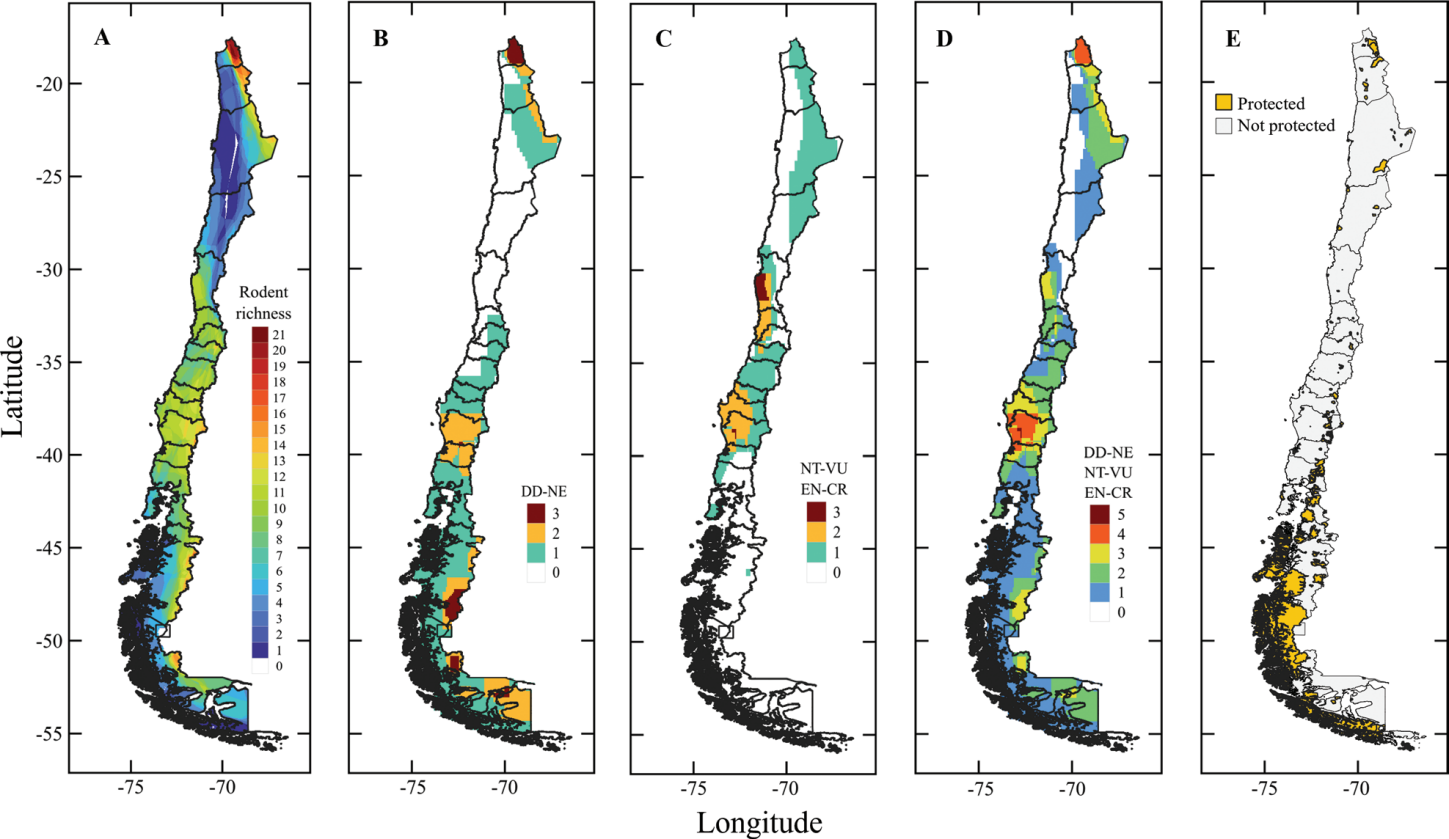
Richness species and threatened status of rodents, and Terrestrial Protected Areas of Chile. **A.** Richness of 64 species rodents of Chile; **B.** Distribution of species considered Data Deficient and Not Evaluated (13 rodent species); **C**. Distribution of rodents classified as Near Threatened, Vulnerable, Endangered and Critically Endangered (8 rodent species); **D.** Distribution of rodents classified as Data Deficient, Not Evaluated, Near Threatened, Vulnerable, Endangered and Critically Endangered (21 rodent species) (Jetz et al. 2012, IUCN 2024a); **E.** Distribution of Protected Terrestrial Areas of Chile (BCN 2024).

We found peaks of species richness in northern (*n* = 21 species, 17–22 °S) and south-central Chile (*n* = 38, 37–45 °S). Rodent species classified as “data deficient” and “not evaluated” (*n* = 16) clustered in northern (17–25 °S), central (32–38 °S), and southern Chile (42–55 °S), whereas the species classified as “threatened” (*n* = 9) occurred in the northern and central portions of Chile (Fig. 3; Suppl. material 1: table S2). Protect areas were distributted among sites where there is lower richness and number of threatened rodent species (Fig. 3).

**Figure 4.**
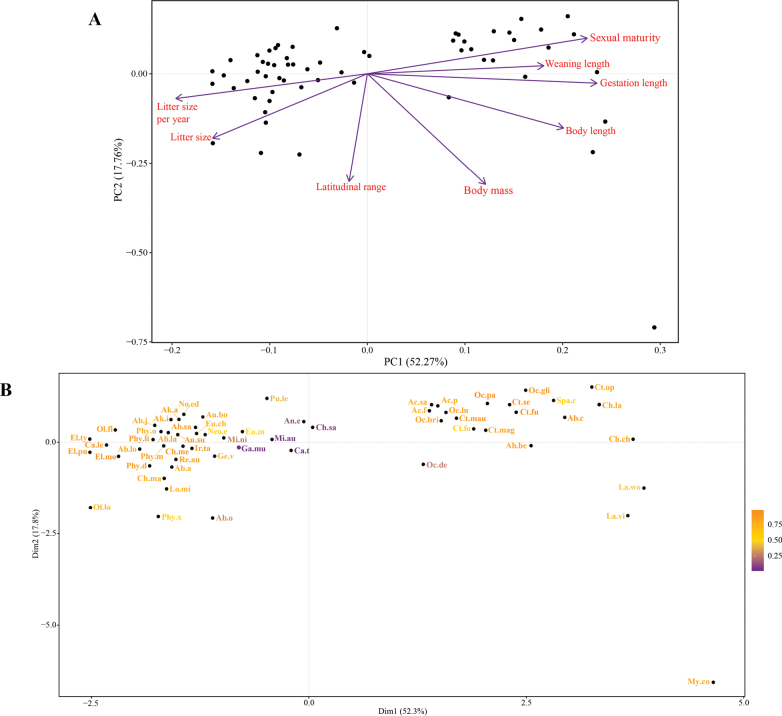
Principal Component Analysis (PCA) of the ecological and biological traits of the evaluated species. **A.** Biplot diagram of the PCA showing the distribution of 60 species based on the first two principal components, PC1 (52.27%) and PC2 (17.76%). The arrows represent the direction and magnitude of the variables included in the analysis and dots represent the distribution of rodent species; **B.**PCA shows the quality of species representation in space, coded with a color scale ranging from yellow to purple, reflecting how well the species project themselves in that area.

Trait data revealed clustered and isolated species through the trait space (Fig. 4). Traits that most influence the species assemblage along principal component 1 explained 52.3% of trait variation, including gestation length (0.46), sexual maturity (0.44) and body length (0.39), which were positively associated. Principal Component 2 explained 17.8% of trait variation. Variables that have the greatest influence on component 2 included body mass (-0.60), latitudinal range (-0.59), and litter size (-0.35), all negatively associated (Suppl. material 1: table S5). Species distributed along the first principal component tended to differ according to gestation length and sexual maturity, while along the second axis, species were differentiated by body mass and latitudinal range. The rodent species with the greatest contribution to the trait variation included *Myocastor coypus*, *Lagidium viscacia*, *Lagidium wolffshoni*, *Chinchilla chinchilla*, and *Ctenomys opimus* (Fig. 4B).

## ﻿Discussion

### ﻿Distribution

Chile is a country of complex geomorphology influenced by unique biogeographical features such as the Andes mountain range to the east, the Atacama Desert to the north, the Pacific Ocean to the west, and the icefields, fjords, and channels to the south (MMA 2018). Chile also has an extreme geographic length of 4300 km, an average width of 180 km, and a range of elevations of 0–6893 m a.s.l., generating diverse ecoregions and climatic zones (Peel et al. 2007; Schutz 2015; Sarricolea et al. 2017; MMA 2018). Despite a modest terrestrial-mammal diversity contrasting with megadiverse neighbor countries (e.g., Peru, Bolivia), Chile’s 166 species of native mammals have a moderated endemism (12% endemic species) (D'Elía et al. 2020; Iriarte 2021).

In Chile the most diverse mammal order is Rodentia with approximately 69 species (D'Elía et al. 2020; Iriarte 2021), which is also the order with most endemic species (*n* = 14 endemic species; Suppl. material 1: table S1; D'Elía et al. 2020). The high endemism of rodents could be explained by geographical factors such as the glacial history and the climatic heterogeneity of Chile (Cofré et al. 2007; Vergara et al. 2014). Also, rodent diversity presents a complex latitudinal pattern that does not correspond to the typical decrease in species as latitude increases (MMA 2018). Instead, the higher richness of rodents in the north and in the center-south portions of the country (Fig. 2A) could be linked to their variability in geographic ranges (Cofré and Marquet 1999; Hernández-Mazariegos et al. 2023). For example, 57 rodent species have restricted ranges (1–10 latitudinal degrees) and 12 species have between medium and large distributional ranges (11–35 degrees; Fig. 1; Suppl. material 1: table S1).

The distributional patterns of some rodent species in Chile have changed over time, with some species expanding and others contracting their ranges. For example *Geoxus valdivianus*, *Irenomys tarsalis*, *Abrocoma bennettii*, *Abrothrix hirta*, and others have experienced shifts in their known distributions (Kelt et al. 2008; Guzmán and Sielfeld 2011; Teta and Pardiñas 2014; MMA 2018). Also, reports of new species (e.g., *A. hirta*, *Abrothrix manni*, *Geoxus lafkenche*, *Eligmodontia dunaris*, *O. yatesi*) and new additions (e.g., *O. flavescens*, *P. lemminus*) are relatively frequent in the literature (Spotorno et al. 2013; Teta and Pardiñas 2014; D'Elía et al. 2015; Teta and D'Elía 2016; Palma and Rodríguez-Serrano 2018; Quiroga-Carmona et al. 2023a, 2023b). Recent species discoveries and reclassifications of rodents make this group complex with respect to its accurate distribution and richness patterns. Vegetation type and environmental factors are associated with rodent species distribution and richness (Muñoz-Pedreros et al. 2010; Vergara et al. 2014; Zúñiga et al. 2021). Small-mammal diversity in Chile is also influenced by ecological, environmental, and historical factors (Cofré and Marquet 1999; Cofré et al. 2007; Vergara et al. 2014; Hernández-Mazariegos et al. 2023). The highest mammal richness in Chile is concentrated in three ecoregions, including the Puna in the north, the Mediterranean and Valdivian Forest in central-south Chile, and the Patagonian Steppe and Forests in the southernmost parts of the country (MMA 2018; Hernández-Mazariegos et al. 2023).

### ﻿Conservation

The current global-biodiversity decline suggests an ongoing sixth mass extinction (e.g., Ripple et al. 2017; WWF 2020; Cowie et al. 2022; Wiens and Saban 2025). For terrestrial ecosystems around the world, the main drivers for the biodiversity decline include habitat destruction, over-exploitation, climatic change, pollution, and biological invasions (Dirzo et al. 2014; IPBES 2019; WWF 2020; Bellard et al. 2022). The importance of each biodiversity threat depends on the taxon, ecosystem, duration of the pressure, and metric considered to measure biodiversity loss (Bellard et al. 2022). For example, in the tropics the most important pressures are habitat loss and overexploitation, while on islands, biological invasions are the major pressures on local biodiversity (Bellard et al. 2022). In Chile, a key threat to biodiversity decline is land use change (e.g., natural areas converted to forest plantation, agriculture, urban areas), with the most perturbated area being the Mediterranean ecoregion of central Chile (Miranda et al. 2017; Benavidez-Silva et al. 2021). Degradation of the Mediterranean ecoregion is relevant in terms of biodiversity conservation because this area is home to a great wealth of rodent species (*n* = 15; Fig. 1). The literature suggests that land use change is reducing native fauna here (Kelt 2001; Zúñiga et al. 2021), as well as local extinction of native rodent species (e.g., *O. bridgesi*, *Aconaemys fuscus*) (Simonetti 1994).

Chile is considered a continental island due to its isolation caused by biogeographic barriers (MMA 2018; Rivera et al. 2023). As such, biological invasions are also a key factor linked to biodiversity loss in continental Chile (MMA 2018). In fact, of the 22 exotic mammal species reported in Chile (D'Elía et al. 2020; Iriarte 2021), at least 14 species are considered harmful to local ecosystems (e.g., cows, cats, dogs, rats, minks; Lobos et al. 2005; Vergara et al. 2014; MMA 2018). Invasive species compete for resources and can also negatively affect the health of local species. For example, pathogen (Canales-Cerro et al. 2022; Cevidanes et al. 2023) and parasite (Poo-Muñoz et al. 2016) transmission from exotic to native wildlife have been reported in central and southern Chile.

In Chile, rodents have been reported to be the most at risk of extinction among mammals species (Gaulke et al. 2019). Currently, we found that of the 69 rodent species found in Chile, nine (13%) are considered at risk of extinction, and 16 (23%) are classified as “data deficient” or “not evaluated” (IUCN 2024a) (Fig. 1; Suppl. material 1: table S2). The number of rodent species in Chile classified at risk of extinction could increase as more data become available (Fig. 1; Suppl. material 1: table S2). Species classified as “data deficient” and “not evaluated” taxa listed in these categories could actually be treated (IUCN Standards and Petitions Committee 2024b). For example, a study used a novel spatial-phylogenetic statistical to provide initial baseline predictions of threat status for data-deficient species determined 331 additional potentially threatened mammals, with elevated conservation importance in rodents (Jetz and Freckleton 2015). Likewise, the number of species lacking information for their classification reveals a deficiency of rodent research from a conservation perspective and potential future research opportunities.

National conservation assessments in Chile demonstrate that some rodent species are at higher extinction-risk classification than what is reported in international assessments (IUCN 2024a; MMA 2024). Emblematic examples include *Cavia tschudii* (Montane guinea pig) listed as “least concern” in an international assessment (Dunnum and Teta 2016) but classified as “vulnerable” in Chile. Similarly, *Galea musteloides* (Common yellow-toothed cavy) classified as “data deficient” internationally (Roach 2016b) was classified as “near threatened” in Chile. Furthermore, *Microcavia australis* (Southern mountain cavy), listed as “least concern” internationally (Roach 2016c), has been categorized as “near threatened” in Chile (Suppl. material 1: table S2) (MMA 2024). In addition, some rodent species have changed their threat classification. For example, *Octodon pacificus* (the Mocha Island degu), an endemic rodent species of Chile, was reclassified from “vulnerable” to “critical endangered” in a 12-year period due to habitat fragmentation (Roach 2016f; Vianna et al. 2017). The discrepancy between international and national classifications may be a challenge for rodent species conservation plans in Chile. The mismatch between international and national classifications of extinction risk could be aggravated with recent taxonomic changes, new reports, and new species. Funding is needed to generate data (e.g., abundance, threats) on Chilean rodents to determine their current conservation status.

Rodent species that remain at risk of extinction include *C. chinchilla* (Short-tailed chinchilla), which has been linked to illegal hunting and trapping to almost drove the species to extinction. Currently, its threats include mining, agriculture, illegal extraction of wild, and a lack of habitat and education (Roach and Kennerley 2016; Gallardo et al. 2021). In the case of the abrothrichine rodent *Geoxus annectens*, listed as vulnerable, its habitat has been fragmented due to increasing pressure from logging activities (Patterson and D’Elia 2018). Additionally, *O. lunatus* (Moon-toothed degu) is listed as near threatened due to loss habitat to agricultural expansion and livestock grazing (Roach 2016e). In general, in Chile the main causes of threats to rodents are agriculture expansion (e.g., livestock farming, logging, hunting, crops), which actually threat at least 22 species (Suppl. material 1: table S3) (IUCN 2024a)

In Chile, the main strategy for biodiversity conservation is the establishment of terrestrial protected areas, where the “Sistema Nacional de Áreas Protegidas” (SNASPE) covers 20.2% of the country’s territory (Marquet et al. 2019). We found, however, that the protected areas in Chile do not align with the richness and distribution of rodent species classified as “data deficient”, “not evaluated”, and “threatened” (Fig. 3). Unfortunately, only approximately half of the protected areas in Chile have management plans and only 14% of these plans are considered ‘efficient’ (Schutz 2015; Petit et al. 2018). Instead, most Chilean territory has limited to null protection, with protected areas largely distributed in isolated, biodiversity-poor regions in the southernmost extent of the country (Petit et al. 2018; Marquet et al. 2019).

More effective biodiversity management is necessary in Chile to better account for species rarity and endemism. For instance, incomplete biodiversity metrics, such as species richness assume that all species has have equal ecosystem function (Rosauer et al. 2009; Lee and Mishler 2014). Improved biodiversity metrics should account among species to integrate the evolutionary history and functional processes of taxa. Examples of revised biodiversity metrics that could help to mitigate perilous ecological assumption include phylogenetic diversity, phylogenetic endemism, functional diversity, and evolutionary distinctiveness (Faith 1992; Isaac et al. 2007; Rosauer et al. 2009; Safi et al. 2011; Veron et al. 2018). Applying these metrics to inform conservation and management plans could help to identify areas and species with the greatest ability to improve conservation efforts (Faith 1992: Hu et al. 2021).

### ﻿Rodent as umbrella species

Small mammals, such as rodents, have high functional diversity and play fundamental roles in ecological processes (Lacher et al. 2016; Formoso and Teta 2019). Rodents maintain ecosystem function and services and are indicators of ecosystem health (Wan et al. 2022). For example, characteristics such as short life expectancy, high reproductive capacity, high diversity, and wide geographic distribution make rodents able to respond rapidly to global change (Wan et al. 2022). In this sense, our PCA analysis reveals that the greatest variance from the traits evaluated were linked to reproduction (i.e., gestation length, sexual maturity, litter size; Fig. 4, Suppl. material 1: table S4). This suggests that reproductive traits are important in the life dynamics of Chilean rodents and can be considered key predictors for monitoring populations in the context of global change (Lima et al. 1999; Zúñiga et al. 2021).

Following the trait analysis, species with rapid life cycles and high reproductive capacity (i.e., high values of litter size and number of litters per year) (e.g., *Abrothrix olivacea*, *M. coypus*, *Octodon degus*, *Eligmodontia puerulus*, *Abrothrix longipilis*, *Oligoryzomys longicaudatus*; Suppl. material 1: table S3) could be used as key to their ecosystems (sentinel species) and rapid response to environmental changes. Likewise, species with high reproductive rates and wide distribution range (e.g., *Loxodontomys micropus*, *A. olivacea*, *O. longicaudatus*; Suppl. material 1: table S4) and abundance (e.g., *Phyllotis darwini*, *G. valdivianus*, *I. tarsalis*, *A. longipilis*, *O. longicaudatus*, *L. micropus*; Vergara et al. 2014) could be key to monitor vegetation regeneration and dispersal of plants (e.g., *Chusquea valdiviensis* and *C. quila* in Chile) (Holz and Palma 2012; Vergara et al. 2014). Nevertheless, given the characteristics of each species, functional diversity derived from out trait assessment revealed that conservation approaches must be species-specific. Some species traits have shown to be predictors of extinction risk for many taxa (Chichorro et al. 2022). For example, ecological traits considered as predictors of extinction risk include habitat breadth and geographic range size (Ripley et al. 2017). Other traits that may be universal predictors include offspring size, fecundity, generation length, and altitudinal range, which can be combined with taxon-dependent traits as body size, diet breadth, trophic level, and microhabitat (Chichorro et al. 2022). Following our result, the traits that best explained the greatest variance could also be interpreted as good predictors of extinction risk and uniqueness (Fig. 4, Suppl. material 1: table S5).

### ﻿Ecosystem services

Rodents have several ecosystem services, such as dispersers of seeds and fungi, pollinators, modifiers and facilitators of vegetation change, soil aerators, and prey of other vertebrates (e.g., cats, foxes, raptor, reptiles) (Vergara et al. 2014; Lacher et al. 2016; Zoeller et al. 2016; Godó et al. 2022). Rodents have commercial uses that include food, textile, pets, and some species utilized for laboratory research (Lacher et al. 2016; Kennerley et al. 2021). These diverse ecological roles and economic uses highlight the importance of rodents for both ecosystem functioning and human society.

In the southern cone of South America, rodents were a food source for humans in the Andean Mountains of central Chile (e.g., *O. degus*, *A. bennettii*; *Spalacopus cyanus*) (Simonetti and Cornejo 1991). Similarly, in Tierra del Fuego Island the Selk’nam indigenous people consumed *C. magellanicus* rodents due to their size and conspicuousness (Borrero 1979; Gusinde 1990; Pardiñas 1999; Jaksic 2023). Currently, many cultures in South America consume rodents as a source of food (Lacher et al. 2016). For example, in the Andes region 64 million of guinea pigs (*Cavia porcellus*) are raised for consumption each year, and in Amazonia the agoutis (*Dasyprocta leporina*) and pacas (*Cuniculus paca*) are approximately 40% of the game consumed by indigenous people each year (Lacher et al. 2016).

Rodents are also popular as pets, with guinea pigs, chinchillas (*C. chinchilla*), and other South American rodent species being commercialized in a large pet industry (Lacher et al. 2016). In natural reserves of South America, charismatic and endemic rodent species also gather tourist attention, such as *C. chinchilla*, *L. viscacia*, *M. coypus*, and *O. degus* in Chile (Bernal 2016; Ojeda et al. 2016; Roach 2016d; Roach and Kennerley 2016), and *Dolichotis patagonum* in Argentina (Roach 2016a).

### ﻿Zoonoses

Climate and anthropogenic pressure are key drivers of rodent distribution (Loyola et al. 2012). Due to their sensitivity to environmental changes, rodents can act as indicator of ecosystem alteration, serving as early warning signals for climate change, conservation challenges, and shifts in biodiversity (Lima et al. 1999; Zúñiga et al. 2021; Wan et al. 2022). This is relevant from a public health perspective because rodent abundance, which is linked with high reproductive rate, has been linked to zoonotic viruses (Meerburg et al. 2009; Tian et al. 2015).

Rodents are hosts for at least 60 zoonotic pathogens caused by a broad taxonomic range of pathogens (e.g., viruses, bacteria, helminths, protozoa, fungi) (Han et al. 2015; Dahmana et al. 2020; Jamil et al. 2021). In Chile, both native and invasive rodent species serve as hosts for a plethora of zoonotic parasites and pathogens, including endoparasites like *Trypanosoma cruzi* and *Hymenolepis* sp. (Yefi-Quinteros et al. 2018; Riquelme et al. 2021), and viruses such as hantavirus (Torres-Pérez et al. 2019).

A relevant rodent-borne emerging infectious diseases in Chile is Andes orthohantavirus strain (ANDV), which in humans causes hantavirus cardiopulmonary syndrome (HCPS) (Torres-Pérez et al. 2004, 2019; Medina et al. 2009). Andes orthohantavirus is among the most important emerging pathogens of pandemic potential (Khan et al. 2021). The main host of ANDV is the sigmodontine rodent *O. longicaudatus* (Palma et al. 2012). Andes orthohantavirus has also been reported in other sigmodontines, at a lower incidence, in species such as *A. olivacea*, *A. longipilis*, *A. hirta*, *A. sanborni*, *P. darwini*, and *L. micropus* (Torres-Pérez et al. 2019). Human ANDV cases reported are linked to the south-central areas of the geographic distribution of rodents in Chile (30–40 °S) (Palma et al. 2012; Astorga et al. 2018; Torres-Pérez et al. 2019).

A different strain of hantavirus, the Seoul strain, has been reported in invasive species in Chile (e.g., *Rattus norvegicus* and *Rattus rattus*, Muridae family; Lobos et al. 2005; Torres-Pérez et al. 2019). Seoul orthohantavirus can cause hemorrhagic fever and renal syndrome with a low mortality (1%) in humans (Hart and Bennett 1999). Also, in *O. flavescens* have been diagnosed antibodies to hantavirus in Uruguay (Delfraro et al. 2003). However, the potential participation of new (e.g., *A. manni*, *O. yatesi*; D'Elía et al. 2015; Palma and Rodríguez-Serrano 2018) and suspected rodent species (e.g., *O. flavescens*, Quiroga-Carmona et al. 2023b) in hantavirus transmission in Chile remains unknown (Brennan et al. 2024).

Several studies have shown that ecosystems with greater rodent diversity can exhibit lower pathogen prevalence compared to those with low species richness (Ostfeld and Keesing 2000; Schmidt and Ostfeld 2001; Suzán et al. 2009). This phenomenon, known as the ‘dilution effect,’ has been widely documented in the case of Lyme disease, where higher diversity of tick hosts reduces pathogen transmission by diluting the influence of highly competent reservoirs, such as for the white-footed mouse (*Peromyscus leucopus*) (LoGiudice et al. 2003; Keesing et al. 2006). From a conservation perspective, identifying areas with high or low rodent species richness and understanding their role as pathogen reservoirs underscores the importance of preserving their biodiversity. Paradoxically, species loss could increase the risk of disease rather than mitigate it, reinforcing the ecological value of maintaining diverse rodent communities. Moreover, this approach could help mitigate the negative perception of the order Rodentia by highlighting its key role in ecosystem stability and health.

### ﻿Research opportunities

Rodents are commonly considered pests to human societies but they play an important role in maintaining ecosystem function, services, and are good indicators of ecosystem health (Wan et al. 2022). Rodents could be a model group to measure biodiversity resilience to environmental change by combining evolutionary history and functional diversity with efforts to mitigate global change. Rodents could also be used to design and evaluate modern strategies to identify priority species, sites for biodiversity conservation and resilience, and design conservation strategies for protected areas. For improved rodent conservation it is important to integrate ecological, evolutionary, and biogeographic patterns, as well as epidemiological data (Hidasi‐Neto et al. 2015; Bovendorp et al. 2019).

Furthermore, the discovery of new rodent species, changes in distribution, and taxonomic revisions may help to understand the circulation and maintenance of some zoonotic diseases (e.g., *O. yatesi* and *O. flavescens*; Palma and Rodríguez-Serrano 2018; Quiroga-Carmona et al. 2023b). For example, the reclassification of a wide distributed rodent species in Chile such as *A. longipilis* allowed the finding of a different species such as *A. hirta* (Teta and Pardiñas 2014), from 35 °S to the north of Tierra del Fuego, thus explaining the occurrence of ANDV in the southernmost portion of South America (Torres-Pérez et al. 2016). Also, human-dimension science is a fertile soil in rodent research to better understand how human perception drives biodiversity loss, which can be used to inform future conservation efforts. Similarly, it is unclear what the perspective of people is about native rodents in Chile, which limits opportunities to advance wildlife management and public health.

## ﻿Conclusions

The diversity patterns reported here reflect the ecological and geographic complexity of rodents of Chile and underline the need for conservation approaches adapted to the species-rich areas. The discrepancies between national and international classifications of extinction risk indicate that effective monitoring and open data are needed for more accurate estimates of threats to local biodiversity. Changes in the geographic distribution and new records of rodent species in Chile have implications in biodiversity conservation, ecology, evolution, and epidemiology.

## ﻿Additional information

### Author ORCIDs

Wendy C. Hernández-Mazariegos https://orcid.org/0000-0003-3176-3281

R. Eduardo Palma https://orcid.org/0000-0002-0806-1268

Luis E. Escobar https://orcid.org/0000-0001-5735-2750

### Data availability

All of the data that support the findings of this study are available in the main text or Supplementary Information.

## Supplementary materials

Supplementary material 1 Additional information Data type xlsx Explanation note **table S1.** Endemism and distributional ranges of Chilean rodents. **table S2.** Conservation and protection status per rodent species of Chile. **table S3.** Rationale of threat status of rodents of Chile. **table S4.** Biological traits of Chilean rodents. **table S5.** Factor loadings of the Principal Components Analysis of the biological traits of Chilean rodents. https://binary.pensoft.net/file/1427581This dataset is made available under the Open Database License (http://opendatacommons.org/licenses/odbl/1.0/). The Open Database License (ODbL) is a license agreement intended to allow users to freely share, modify, and use this Dataset while maintaining this same freedom for others, provided that the original source and author(s) are credited.Wendy C. Hernández-Mazariegos, R. Eduardo Palma, Luis E. Escobar
